# RNA Helicase Vasa as a Multifunctional Conservative Regulator of Gametogenesis in Eukaryotes

**DOI:** 10.3390/cimb45070358

**Published:** 2023-07-05

**Authors:** Vladimir E. Adashev, Alexei A. Kotov, Ludmila V. Olenina

**Affiliations:** Department of Molecular Mechanisms for Realization of Genetic Information, Laboratory of Biochemical Genetics of Animals, National Research Center “Kurchatov Institute”, Kurchatov Sq. 1, 123182 Moscow, Russia; adashev.vladimir@gmail.com (V.E.A.); kotov_alexei@mail.ru (A.A.K.)

**Keywords:** DEAD box RNA helicase, gametogenesis, nuage germ granules, germline stem cells, liquid biocondencates, sexual dimorphism, piRNA pathway

## Abstract

Being a conservative marker of germ cells across metazoan species, DEAD box RNA helicase Vasa (DDX4) remains the subject of worldwide investigations thanks to its multiple functional manifestations. Vasa takes part in the preformation of primordial germ cells in a group of organisms and contributes to the maintenance of germline stem cells. Vasa is an essential player in the piRNA-mediated silencing of harmful genomic elements and in the translational regulation of selected mRNAs. Vasa is the top hierarchical protein of germ granules, liquid droplet organelles that compartmentalize RNA processing factors. Here, we survey current advances and problems in the understanding of the multifaceted functions of Vasa proteins in the gametogenesis of different eukaryotic organisms, from nematodes to humans.

## 1. Introduction

**Figure 1:**
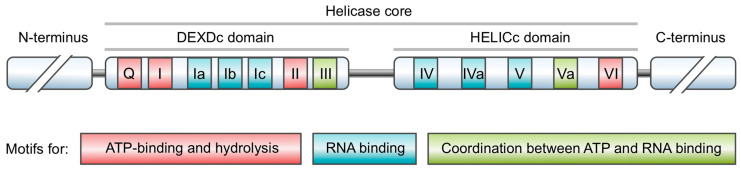
General scheme of DEAD box helicase structure. The following regions are indicated: the N-terminal variable domain; the DEAD-like helicases superfamily (DEXDc) domain; the helicase superfamily C-terminal (HELICc) domain; the C-terminal nonconservative domain. Color bars in the bottom indicate conservative motifs involved in ATP-binding and hydrolysis (red), RNA binding (blue), and coordination between ATP and RNA binding (green). The scheme is modified from [10,11].

The DEAD-box-containing RNA helicase Vasa (DDX4) is a well-known marker of animal germ cells. Using model and non-model organisms, it has been shown that Vasa is involved in germline specification, gamete production, and the piRNA silencing pathway that controls the activity of transposons and other harmful genomic elements in a large number of Metazoan species [1,2,3,4,5,6,7]. The *vasa* gene was initially discovered in *Drosophila* by screening for recessive lethal maternal effect mutations [8,9]. Like all members of the DEAD box superfamily, Vasa has a highly conservative core helicase region of about 400 amino acids, which contains at least 12 characteristic motifs in conservative positions, forming two RecA-like domains, the N-terminal DEAD-like helicase domain (DEXDc) and the C-terminal helicase superfamily domain (HELICc) (Figure 1). These domains bind both ATP and RNA and provide RNA helicase and ATPase activities [10,11]. The DEAD box designation for the superfamily originated from the amino acid sequence of the conservative catalytic site (DEAD) which is a part of motif II, also referred to as the Walker B motif. The core helicase region of Vasa is able to bind a six-nucleotide fragment along the sugar–phosphate backbone of target RNAs [12]. During the unwinding of double-stranded RNA duplexes, DEAD box helicases undergo a series of conformational rearrangements. The co-binding of RNA and ATP results in a significant conformational change in the helicase, bringing the RecA-like domains together in a closed state. Closing the interdomain cleft bends the RNA duplex due to steric hindrance with the help of a wedge-shaped helix formed by motif Ib. It leads to local denaturation of RNA strands. After the release of the first strand of RNA, ATP hydrolysis occurs. The subsequent release of phosphate is the rate-limiting step in the RNA unwinding cycle [13,14]. The N- and C-terminal amino acid sequences of Vasa proteins in different organisms vary considerably. Helicases containing the DEAD box are involved at all stages of cellular RNA metabolism.

Understanding the molecular functions of Vasa is crucial for elucidating important aspects of developmental biology. Here, we survey the different Vasa functions that have been revealed in the germline of model and non-model organisms over the past years. Despite the observed diversity of functional manifestations in different organisms, Vasa is considered a basic conservative factor that is necessary for the process of gametogenesis and, thus, for the maintenance of multiple eukaryotic species from generation to generation.

## 2. Vasa in Germ Cell Specification

There are two main mechanisms of germ cell specification in eukaryotes: preformation and induction [15]. Preformation is defined as a mechanism for germ cell specification driven by the transfer of maternally inherited molecular determinants in the form of ribonucleoprotein (RNP) granules. The RNP granules segregate from the embryonic cytoplasm at the posterior end and ensure the subsequent germline fate of primordial germ cells (PGCs) [15,16]. *Drosophila* is one of those animals that inherit maternal germline determinants through the preformation of PGCs. The preformation of PGCs also occurs in many vertebrates, such as the teleost fish *Danio rerio*, the tailless amphibian *Xenopus laevis*, and birds, as well as in the invertebrate worm *Caenorhabditis elegans* [15,17,18,19].

**Figure 2:**
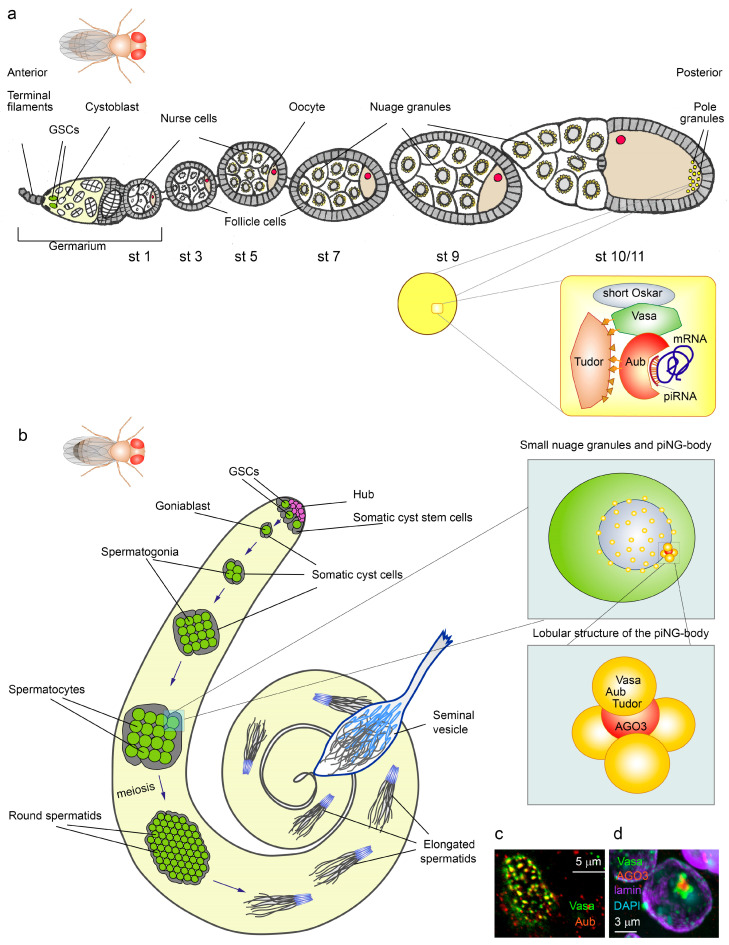
Vasa-positive germ granules in *Drosophila* gonads. (**a**) The scheme of *Drosophila* oogenesis and germ granule distribution. Each ovary contains 12–16 ovarioles. An individual ovariole is shown at the top. At the anterior end of the ovariole, the germarium structure maintains two or three germline stem cells (GSCs) (green ovals). The division of GSC produces a new GSC and a cystoblast. The germ cell cysts arise from cystoblasts that undergo four synchronous divisions. Each ovariole consists of developing egg chambers which comprise an oocyte (light brown) and 15 polyploid nurse cells, connected to each other and surrounded by a monolayer of somatic follicle cells (gray). The nuclei of nurse cells are surrounded by an irregular network of nuage germ granules (yellow dots around nuclei). At the 10/11 stages of oogenesis, pole plasm is assembled and pole granules (yellow dots) are concentrated at the posterior pole of the developing oocyte. RNA helicase Vasa is detected throughout oogenesis flies in high concentrations in the perinuclear nuage granules of nurse cells, as well as at the posterior end of the developing oocyte in pole granules. At the bottom: pole granule organization. Pole granules assemble through phase separation of core proteins and associated mRNAs at the posterior pole of the oocyte. Within pole granules, short isoforms of Oskar, Vasa, Tudor, and Aubergine (Aub) proteins are enriched and provide recruiting mRNAs in piRNA-mediated and piRNA-independent modes. Tudor consists of 11 Tudor domains, which are able to bind symmetrically methylated arginine residues found in Aub and Vasa (orange circles). (**b**) Left: general scheme of spermatogenesis in *Drosophila.* Hub cells at the apical tip of the testis function as a niche to support two populations of stem cells, germline stem cells (GSCs) and somatic cyst stem cells, through the signal molecule secretion. GSCs divide with production of a self-renewal GSC and goniablast; the latter is surrounded by two somatic cyst cells and additionally undergoes four mitotic divisions with incomplete cytokinesis. After that, the cysts of 16 germ cells switch to the spermatocyte program. Spermatocytes enter meiosis, forming 64 haploid round spermatids. At the end, the elongated spermatids move to the basal end of the testis. Mature individual spermatozoa enter the seminal vesicle. Right top: Spermatocyte (green oval) contains multiple small nuage granules (yellow dots) forming an irregular network round the nucleus (light gray) and a large nuage granule, the piNG-body, one per cell. Right bottom: Lobular structure of the piNG-body and mutual arrangement of Vasa, Aub, Tudor, and AGO3 within the piNG-body are shown. Vasa, Aub, and Tudor occupy the peripheral lobes of the piNG-body, whereas AGO3 protein is located in the piNG-body core. (**c**) Small nuage granules around the nuclear surface of spermatocyte. A confocal slice presenting the external surface of spermatocyte nucleus is shown. Significant colocalization of Vasa (green) and Aub (red) signals in the nuage granules producing yellow color is observed. (**d**) AGO3 (red) is located in the central lobe of the piNG-body and colocalizes with Vasa (green) only along the boundary line regions. The 3D immunofluorescent image of spermatocyte nucleus with the piNG-body on its surface is shown. (**c**,**d**) The images are reproduced from [26] by permission of MBoC under Creative Commons-Noncommercial-Share Alike 4.0 Unported license (https://creativecommons.org/licenses/by-nc/4.0/).

Pole cells (PGC precursors) in *Drosophila* are specified at the syncytial stage of embryonic cleavage, and their specialization is determined by the set of maternal proteins and transcripts that accumulate in the pole granules of the developing oocyte [20]. Mutations in the genes encoding the components of the pole granules disrupt the development of gonads and make the progeny of mutant female flies infertile. This phenomenon is known as the *grandchildless* effect. The products of many *grandchildless* genes are necessary for the correct assembly of the pole plasm. Among them, proteins Oskar (its short isoform) and Vasa are considered to be the most significant factors because, in their absence, pole granules are not organized, and developing embryos are not able to form posterior structures and specify PGCs [21,22,23,24]. The maternal protein Vasa accumulates in the pole granules at the posterior end of the developing oocyte starting from stage 10 of *Drosophila* oogenesis (Figure 2a). The recruitment of Vasa is dependent on the advanced location of short Oskar at the posterior pole of the oocyte after stage 9 [21,25].

Mammalian germ cell determination appears to be dependent on inducible cellular interactions during embryonic gastrulation rather than the transmission of cytoplasmic maternal factors. It is known as the induction process of germ cell specification [15,27]. For example, in mice, PGCs are formed from the proximal area of the epiblast near the extraembryonic ectoderm approximately 6.25 days after fertilization at the stage of early gastrulation [28,29]. The first factors of PGC specification found in mice are the transcription factors BLIMP1, PRDM14, and AP2-gamma. It is thought that a variety of signaling events combine to trigger the expression of these factors. Among them is the secretion of BMP4 signaling protein from nearby extraembryonic ectoderm cells and the simultaneous expression of WNT3 factor in the rear visceral endoderm of the epiblast [30,31]. Together, BLIMP1, PRDM14, and AP2-gamma promote the expression of certain germline-specific genes, such as *Nanos3*, and downregulate genes involved in somatic differentiation, such as those of the *Hox* family [30]. By day 7.5 of mouse embryonic development, at the stage of late gastrulation, complementary processes of repression of the somatic program and induction of germline genes lead to the establishment of 30–40 PGCs, which subsequently migrate to the genital ridge [32]. In mice, the zygotic Vasa/MVH protein begins to be expressed in migrating PGCs and exhibits sexual dimorphism in germ cells during gametogenesis [33,34,35,36]. The onset of MVH expression in mammals is comparable to the timing of zygotic expression of Vasa in *Drosophila* [37]. MVH and another DEAD box RNA helicase, GRTH/DDX25, are associated with perinuclear RNP granules in early germ cells of mouse males and are required for spermatogenesis and male fertility [15,35,38,39]. No matter what mechanism of germ cell specification is defined, germ cells in gonads can be identified by their specific expression of Vasa proteins.

## 3. The Role of Vasa in the Structure and Dynamics of Germ Granules

According to recent pioneering studies, germ granules, or nuage, are thought to form as biomolecular liquid-like condensates through phase separation from the remainder of the cytosol [40,41,42]. They lack a membrane-like physical barrier enclosing their content from the outside environment. Phase separation can occur when interactions between macromolecules (proteins and nucleic acids) are thermodynamically more favorable than interactions between these molecules and their solvent (e.g., cytosol). As a result, the molecules and the solvent separate into a condensed phase with a smaller volume and a dilute phase with a bigger volume. Thus, biomolecular condensates partially exclude the aqueous phase [43,44]. The phase separation of proteins is mainly due to weak electrostatic, hydrophobic, π–cationic, cation–cationic, and π–π interactions between neighboring molecules and can be achieved through the interaction of certain domains and the presence of extended regions of intrinsic disorders [43,45]. Intrinsically disordered regions (IDRs) have no defined tertiary structure but contain low-complexity amino acid sequences that can participate in the formation of dynamic multivalent networks that appear in vivo and in vitro as phase-separated droplets [42,43,44]. In addition, the phase separation of proteins can be modulated by post-translational modifications of proteins [46,47], interactions with nucleic acids, and changes in their physicochemical environment. The constituents of the liquid-like condensates are mobile and capable of rapid exchange with the cytoplasm [44].

Recent studies have shown that both prokaryotic and eukaryotic cells use phase separation mediated by DEAD-box-containing helicases with intrinsically disordered terminal domains to compartmentalize various RNA processing reactions. This represents an example of a conservative intracellular organization that has been maintained throughout evolution, from bacteria to humans [48]. A spontaneous condensation of monomers of human homologue Vasa, DDX4, into protein aggregates occurs upon transfection of the culture of somatic cells by *DDX4* carrying construct. The number of aggregates and their sizes are regulated by the available concentration of the free protein monomers, whereas methylation of arginine residues in DDX4 RGG motifs, a reduced ionic strength, and an increase in temperature can lead to reversible dissociation of these structures [42]. It has been shown that the N-terminal IDR and RNA helicase domains of *Bombyx mori* Vasa are responsible for self-association and RNA binding, and all of them are required for liquid-like droplet formation in vitro and for nuage Vasa-body assembly in vivo [49]. DEAD box RNA helicases are thought to broadly control the formation and dynamics of phase-separated RNP granules. In the ATP- and RNA-bound state, these helicases form multivalent interactions with RNAs, which promote the phase separation of these complexes from the cytosol. ATP hydrolysis causes the release of target RNAs from complexes with helicases, thereby disrupting multivalence, and, as a result, provokes the translocation of components of the RNA-containing liquid-like condensates [42,48].

**Figure 3:**
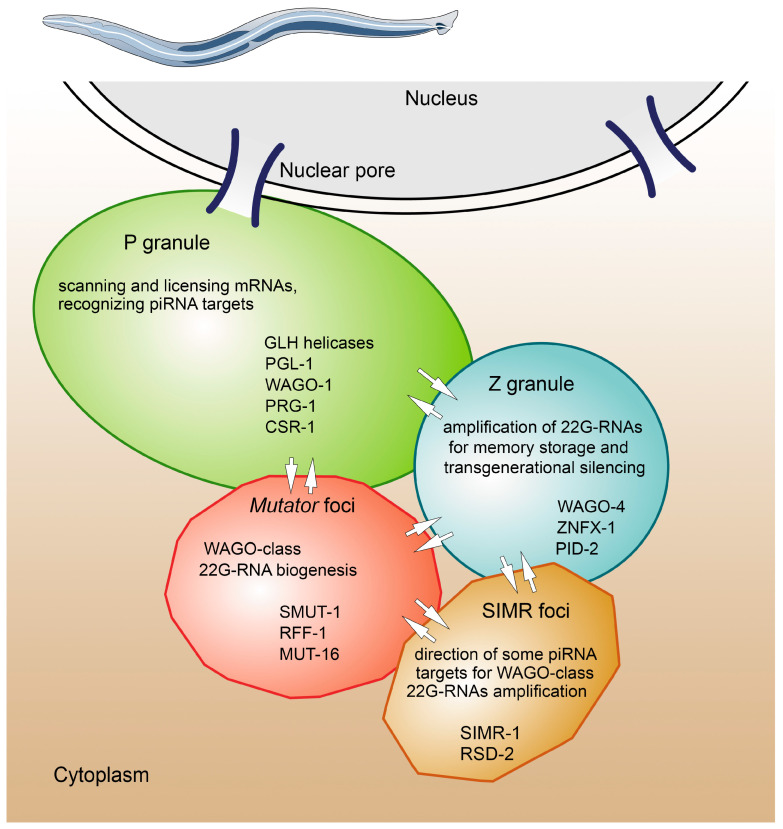
Separation and compartmentalization of germ granules in the *C. elegans* germline. P granules scan nascent transcripts exiting from the nucleus via nuclear pores. P granules form with the aid of GLH helicases (homologues of Vasa) as liquid biocondensates containing CSR-1, PRG-1, and WAGO-1 ARGONAUTE proteins and their associated small RNAs, which can sort foreign transcripts from ones licensed for expression. Z granules provide the amplification of 22G-RNAs for memory storage and transgenerational silencing. *Mutator* foci are sites of WAGO-class 22G-RNA amplification. SIMR foci direct specific piRNA targets for 22G-RNA amplification. Two or more unique protein components for each type of granule are presented. The presumed flow of RNAs and proteins across the granules is indicated by white arrows. The scheme is created using data extracted from a number of papers [51,52,53,54,55,56].

The germ granules of nematode *C. elegans* represent a set of individual condensates. These granules contain subdomains with different electron densities, including an electron-dense layer closest to the nuclear envelope and protrusions facing the cytoplasm [50]. There are at least four classes of condensates, each defined by two or more unique components: SIMR foci, Mutator foci, Z granules, and P granules [51,52,53,54,55,56] (Figure 3). The spatial organization of the granules changes dynamically during the development of the nematode. The P granule and Z granule components overlap significantly in PGCs; these granules are completely fused in early embryos and then separate again when reassembled near the PGC nuclei in 100-cell embryos and remain separated in adult germ cells [53,56]. The majority of research has focused on P granule activity as a liquid drop. P granule proteins are rapidly exchanged between the condensate and the cytoplasm and released into the cytoplasm under high temperatures [40]. P granules are formed by GLH helicases (homologues of *Drosophila* Vasa) and PGL proteins containing arginine-glycine-glycine (RGG) repeats [55,57]. Phenylalanine-glycine-glycine (FGG) repeats are abundant in GLH helicases and are also present in nucleoporins surrounding the central channel of nuclear pores [58,59]. FGG-repeat domains of nucleoporins form a reversible hydrogel matrix that works as the permeability barrier of nuclear pores [60]. Similar permeability characteristics are shared by P granules, and it has been proposed that they serve as extensions of the environment of nuclear pores [58].

Numerous nuage granules that form a network around the nuclei of nurse cells in *Drosophila* ovaries (Figure 2a) also contain components of the piRNA pathway, foremost endonucleases of the ARGONAUTE/PIWI family, Aubergine (Aub) and AGO3, and several members of the TUDOR family proteins. Vasa is a basic architectural factor of these granules [6,61]. piNG-body, a large nuage-associated organelle, is found in the perinuclear area of *Drosophila* spermatocytes together with small nuage granules [26] (Figure 2b–d). The piNG-body is considered the center of post-transcriptional piRNA silencing in spermatocytes owing to the compartmentalization and accumulation of components of the piRNA machinery. Vasa, Aub, Tudor, and some other proteins occupy the periphery of the piNG-body, whereas AGO3 is found in the core lobe [26,62] (Figure 2b–d). The piNG-bodies and small nuage granules are able to migrate along the external nuclear surface and rapidly exchange their components with the cytoplasm [63,64,65,66]. Arginine methylation of the amino-terminal domains of Vasa and Aub provided by arginine methyltransferase Capsuleen/PRMT5 is essential for the assembly of the piNG-body [26]. Disruption of Aub methylation leads to its delocalization into the cytoplasm, the absence of the piNG-bodies, and impaired piRNA biogenesis, while a perinuclear localization of Vasa in small nuage granules is preserved [26,46,47]. 

Early electron microscopic studies uncovered that the pole granules form round, membraneless, dense bodies 0.2–0.5 μm in diameter that contain fibrous material, ribosomes, and nucleic acids at the posterior pole of the developing oocyte in *Drosophila* (Figure 2a). These granules accumulate maternally stored mRNAs and proteins, which are essential for the establishment of PGCs during embryonic development [21,22,67,68]. The short isoform of Oskar plays a central role in the assembly of pole granules. Oskar contains IDR between its LOTUS and RNA-binding domains and a short region of low-complexity sequences. Owing to the presence of these domains, the short Oskar is able to form granules in the absence of other components in *Drosophila* and human cell cultures [69]. While Oskar is not conservative beyond Diptera, other germ granule protein components are present throughout the animal kingdom. Among them are the RNA helicase Vasa, the ARGONAUTE/PIWI family member endonuclease Aub, the founder of the TUDOR domain family Tudor (Figure 2a), factors of translational regulation Nanos, Pumilio, Dazl, and others [20]. A physical interaction of the HELICc domain of Vasa with the LOTUS domain of Oskar is shown [11,23]. The complex Oskar-Vasa provides instructive functions in the enrichment of several hundred specific mRNAs at the posterior pole [70,71]. The Tudor protein, possessing 11 Tudor domains, plays a structural role in the pole granule assembly by binding proteins containing dimethylated arginine residues, including Vasa and Aub [46,47]. Methyltransferase Capsuleen function is required for the assembly of pole granules [72,73]. A lack of arginine methylation of Vasa and Aub results in the *grandchildless* phenotype of the offspring, which does not form pole cells and develops into sterile adults. *Drosophila* pole granules appear to exhibit both liquid-like and hydrogel-like properties [20,69].

**Figure 4:**
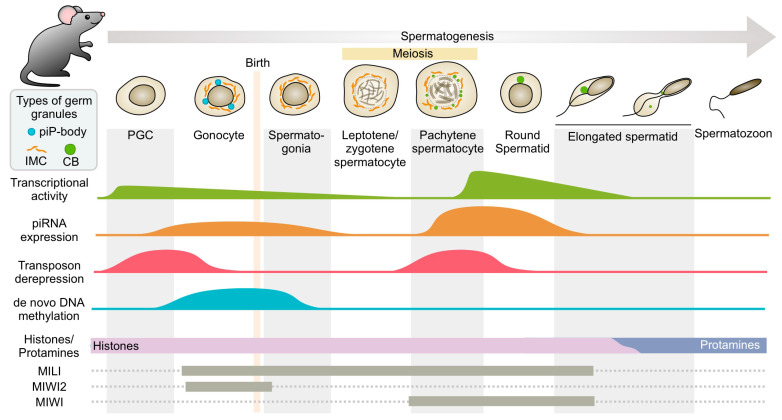
Dynamics of MVH-positive germ granules in mouse spermatogenesis. At the top: two types of germ granules, intermitochondrial cement (IMC) and piP-bodies, are generated in mouse embryonic gonocytes. Both types of granules contain MVH, a murine homologue of Vasa, and distinct components of piRNA machinery. After meiosis in the cytoplasm of round spermatids in adult mice, MVH is concentrated in a single, large, perinuclear, electron-dense granule known as the chromatoid body (CB). Graphs in the middle of the scheme characterize molecular processes in germ cells occurring during mouse spermatogenesis. At the bottom: expression patterns of key proteins of the piRNA pathway, MILI, MIWI2, and MIWI, are shown. The scheme is created using data extracted from a number of papers [74,75,76,78,79,80].

Two types of nuage granules, intermitochondrial cement (IMC) and piP-bodies, were found in mouse embryonic gonocytes, which separately contain key endonucleases of the piRNA machinery and proteins of the ARGONAUTE/PIWI family, MILI and MIWI2, respectively [74,75,76] (Figure 4). Both types of granules contain MVH, a murine homologue of Vasa. After meiosis in the cytoplasm of round spermatids in adult mice, MVH is concentrated in a single, large, perinuclear, electron-dense granule known as the chromatoid body (CB) (Figure 4). It has been found that MVH and the ARGONAUTE/PIWI family endonuclease MIWI are the two major components of murine CB [77]. These findings confirm the crucial role of Vasa in the development and maintenance of mammalian germ granules.

The results obtained on vertebrate and invertebrate organisms suggest that the formation of biomolecular condensates of liquid-like and hydrogel-like types is a conservative principle of the organization of germ granules throughout the animal kingdom, and RNA helicase Vasa is the essential factor for their development and maintenance.

## 4. Sexual Dimorphism of Vasa Functions and Expression in Different Animals

It has been shown that sexual dimorphism in Vasa functions or expression occurs in a variety of model animals and other species. In *Drosophila*, Vasa is the basic component of the pole plasma (or pole granules) accumulated at the posterior pole of developing oocytes. Females homozygous for the *vasa* null allele lay very few embryos, but they do not form germ cells, and adult offspring have abdominal defects [25,81]. However, adult males with the *vasa* loss-of-function mutations remain fertile for a short time after eclosion (Adashev et al., unpublished data). In *Danio rerio*, both males and females need DDX4 for the formation of germ cells and meiosis; however, *vasa* mutants only produce sterile males [4]. Sex-related differences in DDX4 expression during oogenesis and spermatogenesis have also been found for other fish, including medaka, gibel carp, tilapia, catfish, and European sea bass [82,83,84,85,86]. The Asian yellow pond turtle, *Mauremys mutica*, shows a dimorphism in Vasa expression depending on sex. In adult turtles, the level of *vasa* mRNA is much higher in the testes than in the ovaries [87]. In chickens, *Gallus gallus domesticus*, however, male and female embryonic gonads with *DDX4* knockdown contain a reduced amount of PGCs, which confirms the need for Vasa functions to maintain PGCs in embryos of both sexes [88]. 

The mouse Vasa homologue, MVH, is detected in the cytoplasm of premeiotic germ cells in both males and females [89]. In male mice, loss of MVH function causes defects in the proliferation and differentiation of early germ cells into pachytene spermatocytes, and spermatogenesis is arrested at the premeiotic stages from leptotene to zygotene, subsequently leading to germ cell apoptosis [35,89,90]. However, in female mice, impaired expression of the *Mvh* gene does not affect the development of female PGCs, and females homozygous for the *Mvh* mutations are fertile at the same level as wild-type females [35]. MVH, together with MILI, TDRD9, and GASZ proteins, are found in nuage-like aggregates in the cytoplasm of primordial ovarian follicles shortly after birth. However, these aggregates disintegrate during growing and maturing oocytes. In females mutated in *Mvh*, *Mili*, or *Gasz*, there is a decrease in the piRNA production in primordial ovarian follicles, and derepression of retrotransposons is detected. Nevertheless, folliculogenesis appears to be normal in the mutant females, and disruption of the piRNA pathway does not result in sterility. Moreover, wild-type oogenesis is observed even in triple mutants for the *Mvh, Mili,* and *Gasz* genes [91]. Compared to oogenesis, the *Mvh* mutations have a more severe impact on mouse spermatogenesis [89,90]. The stronger regulation of oogenesis can be explained by the obvious differences in the impact of the *Mvh* mutations on spermatogenesis and oogenesis. Thus, in the oogenesis of mammals, as a result of strict selection, only some of the oocytes (10–20%) survive and develop into primordial follicles [92]. In addition, oocytes undergo prolonged arrest at prophase I of meiosis, during which effective repair of damaged sites can occur. Another possible explanation for germline independence from the *Mvh* expression and the piRNA pathway in female mice could be the absence of the fourth member of the PIWI/ARGONAUTE protein subfamily, PIWIL3. PIWIL3 is found in placental mammals but is absent in the *Muridae* family, which includes mice and rats [93]. PIWIL3 may perform some functions specific to female germ cells, as indicated by its high level of expression in bovine oocytes and in human ovaries at the prenatal stage [93,94]. In the absence of PIWIL3, expression of the oocyte-specific isoform of nuclease Dicer, which arose due to the insertion of a retrotransposon into the Dicer gene, was detected in female mice. This insertion ensures the expression of a truncated Dicer variant, which is more efficient in processing double-stranded RNAs into endogenous siRNAs [95]. The functions of MVH and the piRNA pathway in regulating the activity of transposable elements appear to be replaced by Dicer and the siRNA pathway for the passage of mouse oocytes during meiosis [96]. Other undiscovered mechanisms for maintaining oogenesis in the absence of Vasa may also exist, but further study is necessary to clarify them.

## 5. Vasa Functions in *Drosophila*

### 5.1. Multiple Functions of Vasa during Oogenesis

The multiplicity of Vasa functions is studied mostly in *Drosophila melanogaster*. Vasa expression in the germline is essential for embryonic development and PGC specification, as well as for ensuring the process of fly oogenesis [21,97,98]. The RNA helicase activity of Vasa in the ovarian germarium is critical for the continuous development of *Drosophila* germ cells during oogenesis [98,99]. The germarium is the apical region of the *Drosophila* ovary where germline stem cells (GSCs), somatic niche cap cells, and escort cells are found. GSCs divide in a self-renewal mode with the generation of one daughter cell as a stem cell and another cell that leaves the niche and becomes a cystoblast. The cystoblast enters the differentiation niche consisting of somatic escort cells, where it mitotically divides four times, forming a cyst of 16 cystocytes interconnected due to incomplete cytokinesis. The 16 cystocytes are separated into the egg chamber; an oocyte is specified at the basal end of the cyst; and the remaining 15 cells become nurse cells (Figure 2a). The molecular mechanisms of ovarian pathogenesis associated with a lack of Vasa function are the subject of intensive research [97,98,100,101,102]. Atrophy of the germarium, loss of GSCs, cystoblasts, and dividing cysts often occur in females with the *vasa* null mutation. The functions of Vasa in early oogenesis seem to be associated with the maintenance of self-renewing GSC divisions and do not depend on its helicase activity [103]. Only a small number of oocytes in *vasa* null-mutant females complete their development, while most of them degenerate at earlier stages [21,99]. *vasa* mutations also lead to premature reduction in egg chambers in ovarioles with aging [98]. However, the precise molecular role of Vasa in early oogenesis remains poorly understood.

Vasa is enriched in the perinuclear nuage granules of ovarian nurse cells and all premeiotic germ cells of the testes [6,26,61,63] (Figure 2a,b). Nuage granules are centers for post-transcriptional processing of piRNAs and piRNA-mediated silencing of harmful genomic elements, including transposons, genomic repeats, and some protein-coding genes [6,26,61,104]. Being the top hierarchical structural component of nuage, Vasa also facilitates the transfer of long piRNA precursors from the nucleus to nuage [105] and participates in the piRNA amplification cycle (also known as the ping-pong cycle) carried out by Aub and AGO3 proteins for efficient silencing of target transcripts [6,65,66,106,107]. Loss-of-function *vasa* mutations cause the disruption of nuage formation, leading to the dispersion of Aub and AGO3 as well as other components of the piRNA machinery in the cytoplasm and the violation of piRNA-mediated silencing in both the ovaries and testes [6,26,61,104,108]. In the ovaries of *vasa* mutants, a loss of the piRNA biogenesis in nurse cells leads to the activation of multiple mobile elements [6,109], whereas in the testes, transposon mobilization is less pronounced, but protein-coding *Stellates* genes become derepressed in spermatocytes of *vasa* mutant males [26,110,111], causing subsequent meiotic disorders and compromised male fertility [112,113]. Even a transient loss of Vasa expression only between oogenesis stages 2 and 6 leads to transposon derepression in ovarian nurse cells [97]. Mobilization of transposons in female germ cells of *vasa* mutant flies leads to transportation of their transcripts in the oocyte and integration into various loci of the genome. This induces DNA double-strand genomic breaks, leading to severe defects of nuclear chromatin integrity and replication stress, and this can also cause arrest of oogenesis through activation of the checkpoint kinase 2 (Chk2) signaling cascade and subsequent sterility [97,114,115]. It can also result in transposon transfer from mother to offspring embryos, where it can cause DNA breaks, chromatin damage, and the arrest of early embryogenesis [97,116]. The helicase activity of Vasa is shown to be essential for oogenesis and embryogenesis [97].

The localization of Vasa in nuage, as well as the recruitment of Aub and AGO3 in nuage, are independent of Vasa helicase activity, but this activity is required for the piRNA-mediated silencing of target transcripts [98]. The HELICc domain of Vasa is essential for its nuage and pole plasm localization, whereas the N-terminal nonconservative sequence of Vasa is critical for proper pole cell and abdomen formation [11]. In the case of a point mutation in the catalytic core site (DEAD → DQAD), resulting in a permanently closed Vasa conformation, preventing ATP hydrolysis and RNA release, the perinuclear localization of nuage granules is not maintained, they increase in size, and post-transcriptional piRNA silencing is impaired [66,98]. 

### 5.2. Vasa as a Translational Activator

Early studies have shown the involvement of Vasa in the translational activation of *nanos*, *gurken*, and *mei-P26* mRNAs during oogenesis [99,100,101,117,118], although no direct physical interaction of *nanos* or *gurken* transcripts with Vasa has been shown. Vasa directly interacts with the translation initiation factor eIF5B (also known as dIF2) to positively regulate the translation of mRNAs required for oogenesis [100,101,102]. Disruption of Vasa interaction with eIF5B in functional *vasa* mutants results in reduced translation of Gurken, a component of the EGFR signaling pathway, leading to female sterility and phenotypes identical to *vasa* null mutations [100]. Vasa functions as a positive translational regulator of Mei-P26 in vivo by interacting with the U-rich element in the 3′-UTR of *mei-P26* mRNA. Mei-P26 is essential to restrict the expansion and proliferation of germ cells during the early stages of oogenesis. While its expression is modest in GSCs, it is highly expressed in nurse cells of 16-cell cysts. The absence of Mei-P26 expression promotes early germ cell proliferation and aberrant development, which results in a tumor-like ovarian phenotype [119,120]. 

A cohort of translation initiation factors involved in the formation of the pre-initiation complex, eIF2, eIF3, and the eIF4E-4G cap-binding complex, is co-immunoprecipitated with Vasa from *Drosophila* ovaries [24,98]. In addition, it has been shown that Vasa genetically interacts with the translation initiation factor eIF4A. eIF4A is essential for the formation of PGCs in embryos [121]. However, the exact mechanism and many possible targets of Vasa as a translational regulator remain unidentified to date.

It appears that various intracellular Vasa populations are engaged in the regulation of translation and the piRNA silencing. Although it is unclear how this functional separation is governed in germ cells, post-translational modifications of Vasa may be important. Vasa could be a component of a previously discovered network of protein–protein interactions, including Aub, Rm62, PABP, and eIF3 proteins, that have been shown to control germ cell development [122,123].

### 5.3. The Role of Vasa in Pole Granule Assembly

The products of a large number of *grandchildless* genes are required for pole granule assembly in *Drosophila* (Figure 2a). However, the most significant components of the cascade are the products of the *oskar*, *staufen*, and *vasa* genes [21,124]. Vasa is attracted to the pole granules through the interaction of the Vasa HELICc domain (residues 463–621) with the C-terminal extended LOTUS domain (eLOTUS) of the short Oskar isoform [11,23]. Correct localization of Vasa at the posterior pole of the oocyte also requires the presence of an invariant tryptophan residue in the C-terminal position (W660 in *Drosophila melanogaster*), as well as the C-terminal motif 636–646 aa, which is highly conserved among many species and consists of acidic amino acid residues. Deletion of this motif results in an insufficient concentration of pole granules at the posterior pole and the subsequent absence of germ cells in developing embryos [103,125]. The replacement of the C-terminal tryptophan residue with glutamic acid (W660 → E) also leads to a loss of the ability of Vasa to maintain the formation of PGCs and embryonic pattern, disruption of *gurken* translation activation, and significantly reduced activity of Vasa in the piRNA biogenesis [125]. Both Vasa and Oskar are required for the localization and translation of specific mRNAs in the pole granules [20,126,127,128,129,130]. Nevertheless, a direct involvement of Vasa in the activation of their translation is not shown, since translation can be suppressed due to a violation of the assembly of the pole granules themselves. The proteins encoded by these mRNAs are essential for the patterning of embryos and the specification of future germ cells. Translation of *nanos* at the posterior pole of the embryo normally results in the creation of a gradient of morphogen Nanos, while the absence of maternal *nanos* mRNA in pole granules leads to defects in abdominal segmentation of the developing embryo and, in addition, disrupts the migration of pole cells into the developing gonads [128].

### 5.4. Vasa in Drosophila Testes

Despite the fact that Vasa is expressed in germ cells both in the ovaries and testes, the data on its functions in spermatogenesis are rather contradictory. The fertility of young males with *vasa* null mutations have allowed researchers to propose that this gene is not essential for spermatogenesis, which indicates in favor of the sexual dimorphism of Vasa functions in fruit flies [81,131]. However, data about the critical role of Vasa in the formation of nuage granules, piRNA biogenesis, and *Stellate* gene silencing clearly contradict this point of view [26,111,132]. Derepression of *Stellate* genes in the case of piRNA pathway disruption leads to the formation of needle-like Stellate protein aggregates, meiotic disorders, and a decrease in male fertility up to complete sterility [112,113]. Our data also suggest a premature loss of GSCs in the testes of *vasa* mutants (Adashev et al., unpublished data). It remains unclear whether the functions of Vasa in the piRNA pathway and in the maintenance of GSCs are mutually independent. Vasa participates in a wide range of cellular processes, which makes it challenging to learn and comprehend how its competencies are distributed in germ cells.

### 5.5. Vasa Is a Target of the piRNA Pathway in the Gonads of Interspecific Hybrids

**Figure 5:**
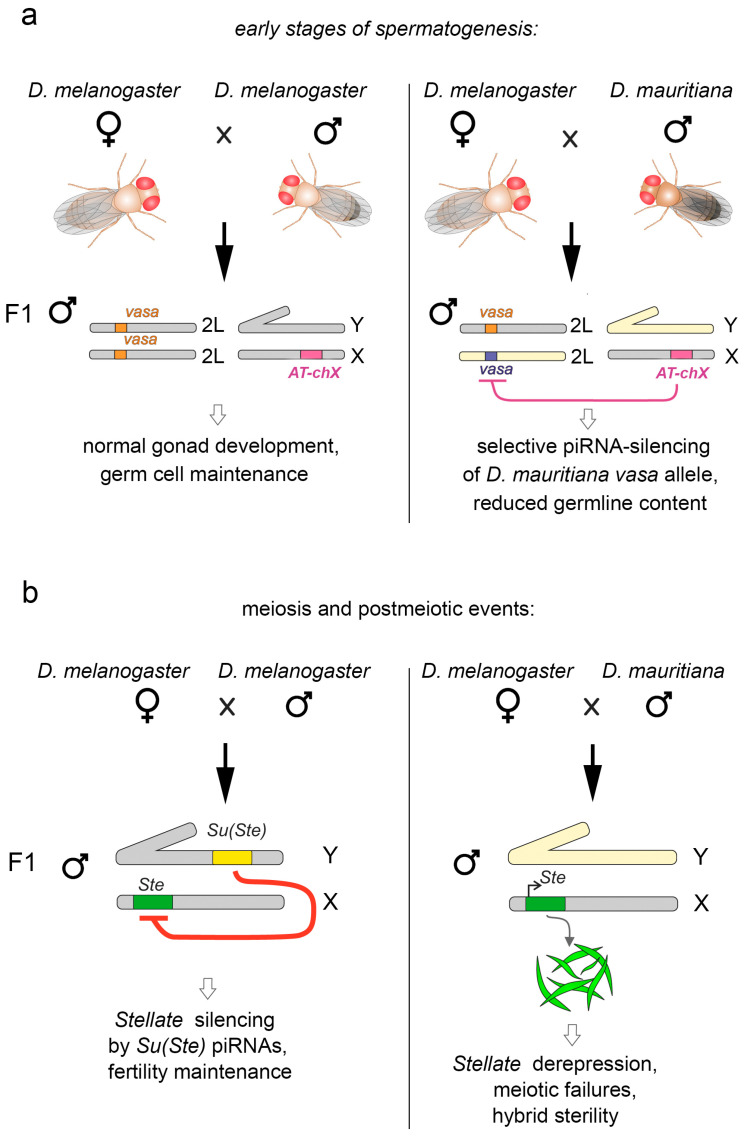
piRNA-dependent misregulation of *vasa* and *Stellate* genes in the testes of interspecies *Drosophila* hybrids. (**a**) piRNA-dependent silencing of the *D. mauritiana vasa* allele in the testes of interspecies hybrids. Silencing of the alien allele of *vasa* by *AT-chX* piRNAs in diploid hybrids appears to cause an insufficiency of Vasa functions in germline maintenance in the early stages of spermatogenesis. (**b**) Derepession of *Stellate* genes in the testes of hybrids lacking the Y-linked *Su(Ste)* locus in the genome causes meiotic failures and male hybrid sterility. Chromosomes of hybrids inherited from *D. melanogaster* are shown in gray, and those from *D. mauritiana* are shown in beige. The schemes are adapted from [133] by permission of Oxford University Press (https://creativecommons.org/licenses/by/4.0/, accessed on 21 February 2019).

Data from a recent study point to the potential participation of Vasa in interspecies reproductive isolation [133]. Earlier, piRNAs that are complementary to *vasa* transcripts were found in the testes of *D. melanogaster* males [134]. However, the biological significance of putative piRNA-mediated downregulation of Vasa, a key germ cell marker, a regulator of gametogenesis and gonad production, has remained unclear for a long time. Multiple repeated genomic regions, *AT-chX* loci, residing in the X chromosome of *D. melanogaster* were later discovered. They are found to be unique for the genome of *D. melanogaster* and to share a homology with two exons of the *vasa* gene [133]. *AT-chX* loci generate a significant amount of piRNAs, being one of the major piRNA clusters in the testes [133,135,136]. However, piRNA-mediated repression of *vasa* via decreasing mRNA or protein product levels is not observed. The identity between the *AT-chX* repeats and the *vasa* sequence (about 76%) does not seem to be enough for effective piRNA silencing. However, the homology between the *AT-chX* loci of *D. melanogaster* and the *vasa* sequences of closely related species *Drosophila mauritiana* (and other fruit flies of the clade *simulans*) is found to be much higher (more than 90%). piRNA-dependent repression in this case should be effective, i.e., the *vasa* allele of *D. mauritiana* potentially could be silenced by piRNAs produced from the *AT-chX* loci. This hypothesis was tested by analyzing testes of *D. melanogaster/D. mauritiana* interspecies hybrids. It is found that only mRNA and protein products of the *vasa* allele of *D. melanogaster*, but not those of *D. mauritiana*, are expressed in the testes of the hybrids (Figure 5a). Presumably owing to the repression of parental allele of *vasa* that are essential for germline development, most hybrid males have reduced testes containing a small number of germ cells and no mature gametes. In the testes of hybrids that possess perfect germline content and normal morphology, strong derepression of *Stellate* genes in spermatocytes is detected due to the absence of the Y-linked *Su(Ste)* piRNA cluster in the hybrid genome (Figure 5b) [133,136]. As previously shown, it disrupts germ cell passage throughout meiosis and subsequent sperm production [112]. Thus, male sterility of hybrid progeny from crossing *D. melanogaster* with closely related species can be caused by at least two circumstances: piRNA-mediated repression of the alien *vasa* allele and derepression of *Stellate* genes (Figure 5). These findings indicate the involvement of the piRNA pathway in reproductive isolation and speciation. Preserving the functionality of its protein product, the *vasa* gene of *D. melanogaster* apparently was subjected to selection pressure and accumulated a sufficient number of nucleotide substitutions to avoid silencing by *AT-chX* piRNAs.

## 6. Functional Homologues of Vasa in *C. elegans*

### 6.1. General Overview of GLH Proteins

The homologue of Vasa GLH-1 (Germ Line Helicase 1) protein is the first identified constitutional component of germ granules, also known as P granules, in the nematode *C. elegans* [1]. Similar to Vasa in *Drosophila*, GLH-1 is part of the protein core of germinal granules, which is conservative in metazoans. Other conservative P granule core proteins include members of the TUDOR and ARGONAUTE families that bind small RNAs. GLH-1 has three paralogs (GLH-2, GLH-3, and GLH-4), but only GLH-1 and GLH-2 contain all domains specific to Vasa proteins [59]. Vasa-defining domains include glycine-rich repeats at the amino end, the helicase core domain, and a carboxy-terminal negatively charged domain. Intrinsic disorder regions, or IDRs, in GLH proteins contain phenylalanine-glycine-glycine (FGG) repeats that facilitate P granule association with nuclear pores [58,59]. In addition to the IDRs and the helicase core, GLH proteins also contain several copies of the retroviral zinc finger (CCHC) type domain [137,138].

### 6.2. Peculiarities of the piRNA Pathway in Nematodes

*C. elegans* are hermaphrodites that undergo spermatogenesis in late larval stages and then switch to oogenesis as adults [139]. All stages of germ cell development are represented in a single animal, which makes *C. elegans* a unique model genetic system. In *C. elegans*, the piRNA pathway is carried out in a different way than it does in *Drosophila* and mammals; 21 nt-long piRNAs (21U-RNAs) enriched with uracil at the 5′ end interact with the PIWI subfamily protein PRG-1. These complexes function in the recognition of specific transcripts, ensuring their accumulation in P granules [140,141,142,143]. The sequences encoding more than 15,000 species of 21U-RNAs are located in two large genomic clusters on the chromosome IV, between protein-coding genes and in introns [143,144]. In contrast to long piRNA precursors needed for piRNA production in mice and flies, 21U-RNAs are transcribed as single transcription units about 25–26 nt long that are then shortened to 21 nt [142,144,145]. Transposons constitute only about 12% of the *C. elegans* genome, and most of them are non-mobile, making this model organism perfect for investigating alternative piRNA functions. While some 21U-RNAs are involved in transposon repression [141], the vast majority of them are complementary to transcripts of protein-coding genes [139,140,146,147,148]. It has been found that PRG-1 interacts with the transcripts of nearly all protein-coding genes expressed during oogenesis, requiring only a partial complementarity of piRNAs with target transcripts, with up to four mismatches being sufficient for the subsequent steps of regulation [146,148]. Although PRG-1 has a conservative catalytic motif DDH and can cut RNA in vitro, it usually does not cut its target transcripts in vivo [148].

The piRNA-dependent binding of PRG-1 to target mRNA triggers the recruitment of RNA-dependent RNA polymerase (RdRP) in association with the protein complex Mutator. This results in the production of endo-siRNAs, also known as 22G-RNAs, from nearby regions of target transcripts [139,147,148,149,150]. piRNA-dependent 22G-RNAs form complexes with WAGO proteins, a class of worm-specific proteins of the ARGONAUTE family [151]. These complexes can be translocated to the nucleus, where they bind to nascent transcripts and carry out epigenetic silencing across multiple generations, providing lysine methylation at position 9 of histone H3 (H3K9) and establishing heterochromatinization of corresponding genomic regions. They can also perform post-transcriptional silencing of mature mRNAs [139,147,148,149]. Only maternal, not paternal, 21U-RNAs are transmitted to the next generation and play an active role in 22G-RNA-mediated transgenerational inheritance. As a result, PRG-1 complexes with piRNAs function as a powerful control mechanism during oogenesis, ensuring transcript licensing for 22G-RNA-mediated silencing. Numerous aberrant 22G-RNAs derived from ribosomal RNA, histone genes, and some other annotated protein-coding genes are produced as a consequence of *Prg-1* mutations. In *Prg-1* mutants, the aberrant 22G-RNAs represent 45% of all WAGO class 22G-RNA reads, but only a very small portion in wild-type animals [152]. Aberrant silencing of histones, ribosomal RNA, and many protein-coding genes in the case of the disrupted piRNA pathway is associated with impaired worm fertility, not immediately but after multiple (up to 50) generations [152,153]. However, to date, it is unclear which molecular mechanisms lead to sterility [152]. Another ARGONAUTE protein, CSR-1, binds to a genetically different class of 22G-RNAs, and this complex is thought to work against the piRNA system and to support the stability of endogenous transcripts in germ cells [147,154,155,156,157].

### 6.3. Functional Features of Vasa-like GLH Proteins

Multiple studies of GLH protein functions in development have demonstrated that loss-of-function *glh-1* mutant worms are viable at 20 °C but become sterile after multiple generations at 25 °C, a phenotype known as the “mortal germline” phenotype [158,159,160]. The loss of both maternal and zygotically encoded GLH-1 results in complete sterility at 26 °C. However, keeping at lower temperatures (16–24.5 °C) restores the fertility of most *glh-1* mutant animals [159]. *glh-4* null mutants show moderate fertility defects. *glh-1*; *glh-2* and *glh-1*; *glh-4* double mutants are practically sterile at any permissive temperature and demonstrate a significant loss or absence of germ cells, as well as an insignificant number of mature spermatozoa or their absence [59,158,159]. The ability for self-renewing division of GSCs is also impaired in *glh-1* mutants [159].

RNA helicase GLH-1 has essential functions in the control of the dynamics and localization of the P granules during the development of *C. elegans* from embryos to adults, and it is implicated in the transgenerational inheritance of the RNAi interference system [160,161]. In early embryos, P granules are cytoplasmic and subsequently concentrated in PGCs. Prior to the first cell division, many P granule components are initially disseminated in the mature oocyte or fertilized embryo before being reassembled into the granules. P granules relocate to the nuclear periphery when zygotic transcription starts during embryogenesis and remain perinuclear almost all the time during germ cell development. In contrast to wild-type embryos, in which GLH-1-containing granules are disassembled during the pseudo-cleavage stage in early embryogenesis, many GLH-1-containing granules remain clearly visible in *glh-1^DQAD^* mutants with impaired ATP hydrolysis. Unlike in wild-type animals, GLH-1 granules in *glh-1^DQAD^* mutants are not sorted exclusively into PGCs but are randomly distributed among somatic and germ cells in early embryos [154]. *glh-1* mutations impair the localization of other P granule components [59,158,159,162]. P granule components become almost completely dispersed in the case of the double mutants *glh-1*; *glh-4* [159,162]. Binding of mRNAs by GLH-1 promotes localization of the transcripts and recruitment of proteins in the P granules, including members of the ARGONAUTE family. This requires the correct functioning of the GLH-1 core domain in the binding and subsequent hydrolysis of ATP in germ cells of both adults and embryos [59,162]. In the germ cells of *glh-1^DQAD^* adult worms, enlarged P granules containing GLH-1 are located in the cytoplasm, while their perinuclear localization is impaired. These enlarged granules also contain CSR-1 and PGL-1. It indicates that the formation of the P granules with the correct localization and component release dynamics requires the RNA-binding activity of GLH-1 [59,162].

*glh* mutant worms show no significant changes in piRNA expression level compared to wild-type animals. However, there is a noticeable reduction in the quantity of 22G-RNAs, both in *glh-1*; *glh-4* double mutants and *glh-1^DQAD^* mutants. This implies that GLH-1 and GLH-4 are not required for piRNA biogenesis but rather promote the production of 22G-RNAs around piRNA-binding sites on target transcripts [55]. Both GLH-1 and GLH-4 facilitate piRNA-induced silencing and function redundantly in this process. The ARGONAUTE proteins PRG-1 and CSR-1 are localized in the P granule condensates on the outer surface of nuclear pores in adult worms (Figure 3) [59,162]. However, the biogenesis of 22G-RNAs, required for piRNA-mediated silencing, is associated with spatially distinct condensates known as the *Mutator* foci [53,150,163]. Earlier, it was unclear how piRNA targeting in the P granules causes the production of 22G-RNA in the *Mutator* foci to carry out transcriptional and post-transcriptional silencing. The *Mutator* foci are often located in close contact with the perinuclear P granules [163]. Recent work has shown that the *Mutator* foci are specifically incorporated into the P granules during spermatogenesis at the pachytene stage [157]. It can be proposed that GLH-1 is a factor that combines the initial targeting of transcripts by PRG-1 with the establishment of WAGO-dependent silencing since the physical interaction of GLH-1 with both PRG-1 and WAGO proteins has been confirmed [59,164]. P granules act as a control point for piRNA-mediated gene silencing, where harmful alien piRNA targets are recognized, while CSR-1 allows maintaining the expression of own essential genes. In loss-of-function *glh-1* mutants, perinuclear P granules are not formed, resulting in the scattering of RNA interference machinery, including PRG-1, WAGO, and CSR-1 proteins, in the cytoplasm. In the absence of a compartmentalized environment achieved in the P granules, hundreds of normally repressed alien transcripts are derepressed, while hundreds of normally expressed own transcripts fail to bind with CSR-1, which results in their repression [55]. These results support the model according to which the binding and release of mRNAs from GLH-1 after ATP hydrolysis provide the influx and release of components that are located in the P granules. In this model, mRNA binding by GLH-1 facilitates multivalent interactions between mRNA, GLH-1, and other RNA-binding proteins that are attracted to the perinuclear P granules. Replacement of aspartic acid residue with glutamine in *glh-1^DQAD^* mutants, which prevent ATP hydrolysis, causes enhanced mRNA binding by GLH-1 in vitro and nonspecific association of ARGONAUTE proteins with 3′ ends of mRNA in vivo [164]. It should be noted that the DQAD mutation of *vasa* in germ cells of insects also leads to increased sizes of germ granules that accumulate Vasa and the PIWI protein AGO3 with impaired dynamics [66,98]. All of these results are consistent with the idea that Vasa-like helicases regulate the dynamics of germ granules in different species in a conservative manner. Thus, according to modern concepts, GLH-1 in worms and Vasa proteins in other species function as solvents for mRNAs in germ granules phase-separated from the cytoplasm [42], providing the accumulation of transcripts and maintenance of them in an unfolded state accessible for their scanning by complexes of ARGONAUTE protein with small RNAs. The functional similarity between GLH and Vasa proteins with respect to the assembly of germ granules is striking, given the features that distinguish the GLH proteins of *C. elegans* from most other Vasa homologues. The FG and FGG repeats are located in the glycine-rich amino-terminal regions of GLH proteins, in contrast to the RG and RGG repeats found in the Vasa protein of *Drosophila* and vertebrates. While RGG repeats presumably mediate the association of Vasa with mRNAs [165], FGG repeats may mediate the association of FGG-rich Vasa homologues with one another or with nuclear pore proteins that are also rich in FGG motifs [58,59]. In addition, all GLH proteins possess several (from two to six) CCHC-type zinc fingers not found in Vasa homologues of *Drosophila* and vertebrates. It is possible that zinc fingers are involved in RNA binding and compensate for the absence of RGG repeats in the GLH family. Note that the ability for self-renewing divisions of GSCs is impaired in *glh-1* mutant worms [159]. This finding supports the conservative functions of Vasa and its orthologues in the maintenance and self-renewal of GSCs, as shown by *Drosophila* studies [21,97,98].

## 7. Vasa (MVH, Mouse Vasa Homologue) in Mice

### 7.1. Dynamics of MVH Expression in the Mouse Testes and the Structural Role of MVH in the Formation of Germ Granules

As previously mentioned, the specification of germ cells in mammals is not dependent on the inheritance of cytoplasmic maternal factors but occurs through the induction mechanism during embryonic gastrulation, when PGCs develop from pluripotent epiblast cells [15,27]. Spermatogenesis in mice is a sequence of events that tightly regulates the differentiation of germ cells in the epithelium of the seminiferous tubules. On the 12th day of embryonic development, during late gastrulation, about 30–40 PGCs migrate through embryonic tissues toward the genital ridge. After that, the following differentiation of PCGs into prospermatogonia (gonocytes) begins on the 12th day of embryonic development [32]. The fetal gonocytes preceding the formation of spermatogonial stem cells enter into a cell cycle arrest up to the second day of postnatal development. Then, on days 9–10 after birth, a part of the gonocytes develop into spermatogonial stem cells, while the rest are involved in the first rounds of spermatogenesis [166]. During the migration and differentiation of PGCs, germ RNP granules emerge in their cytoplasm [167]. There are two types of small germ granules abundant in mouse prenatal gonocytes that differ in their compositions: piP-bodies and intermitochondrial cement (IMC), while after birth the large chromatoid body (CB) starts to assemble in the late pachytene spermatocytes [75,76,78] (Figure 4). The dynamics of the appearance and disappearance of each type of granule have been studied. Thus, piP-bodies are only found in fetal gonocytes, where they are adjacent to IMC granules. IMC granules are found among mitochondrial clusters in fetal gonocytes, postnatal spermatogonia, and pachytene spermatocytes. In late pachytene spermatocytes, CB precursors appear and coexist with IMC granules. At first, they are small and concentrated in the perinuclear space. After meiotic division, IMC granules are no longer detected in haploid round spermatids, but CB is formed as a relatively large single structure adjacent to the nucleus and is detected during subsequent stages of spermatid differentiation, progressively decreasing in size and degrading [77,78]. Multiple protein components of germ granules are conservative across many species, among them DEAD-box-containing RNA helicase MVH (Mouse Vasa Homologue).

Both piP-bodies and IMC granules contain MVH, but the rest of their proteomic composition is distinct. piP-bodies contain components of somatic processing bodies and germ-cell-specific proteins of the piRNA pathway, MIWI2, and TDRD9. IMC granules contain other components of the piRNA pathway, MILI and TRDR1 [75,76]. MIWI2 and MILI are endonucleases of the ARGONAUTE/PIWI family and are key components of the piRNA system, ensuring silencing transposable elements in fetal gonocytes via the ping-pong amplification cycle and de novo DNA methylation of transposable elements in the genome [74,75,76] (Figure 4). TDRD9 and TRDR1 are Tudor-domain-containing proteins. MIWI2 loaded with guide piRNA translocates into the nucleus, where it participates in transcriptional silencing [74]. Thus, piP-bodies and IMC granules contain separate modules of the piRNA machinery, presumably interacting with each other through transient contacts. MVH appears to be an essential structural factor for both types of germ granules in fetal gonocytes.

Due to its relatively large size, CB was first identified under a light microscope in rat testes about 150 years ago [168], and since then it has been the subject of extensive studies. To date, mouse CB composition has been investigated by immunochemical and proteomic methods [77,167]. About 100 proteins have been identified as part of CB [77]. Most of them display RNA-binding activity, while others also participate in RNA metabolism [169]. MVH and MIWI, the third endonuclease of the piRNA pathway, are two major components of CB, together making up about 40% of its proteome. This indicates that CB is functionally involved in the piRNA pathway, and, indeed, transcripts of pachytene piRNA clusters were found in CB content. CB also contains components of the nonsense-mediated RNA decay pathway, RNA-binding proteins associated with translation, splicing complex proteins, proteins involved in microtubule-mediated transport, and other unclassified proteins [77]. Thus, in the testes, MVH is associated with germ granules of all three types, piP-bodies, IMC, and CB, apparently being the main architectural component for all of them. MVH disappears in elongated spermatids during CB degradation [89,90].

Two waves of active transcription are observed during spermatogenesis in mice, which coincide with the appearance of piP-bodies and CB (Figure 4). The first one occurs in the early stages of spermatogenesis, starting in fetal gonocytes and finishing in spermatogonia after birth. This is followed by a significant drop in the level of transcriptional activity during the entry of spermatogonia into meiosis and homologous recombination during meiotic entry (leptotenes and zygotenes) [170]. The next wave of active transcription begins during meiosis and gradually fades during the spermatid elongation stage, which includes chromatin compaction and the replacement of histones by protamines [171,172]. MIWI2 and MIWI expression patterns coincide with the corresponding waves of active transcription: MIWI2 is detected in fetal gonocytes and spermatogonia after birth, and MIWI is expressed at the last stages of meiosis and post-meiotically (Figure 4). Accordingly, prepachytene piRNAs are detected in the MIWI2 expression period, where they are involved mainly in suppressing the activity of mobile elements such as *LINE1* and *IAP* [74], while pachytene piRNAs are detected in the MIWI expression period. The role of pachytene piRNAs in gametogenesis was mysterious until recently.

### 7.2. MVH Is Essential for Proper Spermatogenesis and the piRNA Pathway

Homozygous mutants for *Mvh* develop normally upon reaching adulthood and exhibit normal sexual behavior, but males become sterile [35]. Adult *Mvh* mutant males lack post-meiotic germ cells entirely, and their testes are underdeveloped (consisting of 4/5 of their wild-type size). At the same time, the testes of mice with the *Mvh* mutation at the age of two days do not differ morphologically from the heterozygous control, except for a slight decrease in the size of the seminiferous tubules. However, it has been shown that in the testes of *Mvh* mutant mice, apoptosis events in germ cells at the premeiotic stage occur 10 times more frequently compared to the heterozygous control. In addition, mutant males were found to have a low frequency of PGC proliferation during embryonic development [35]. There is much data suggesting that MVH is essential for spermatogenesis mainly due to its involvement in the piRNA pathway: first, as a direct participant in the piRNA biogenesis, and, second, as a factor that forms germ granules where this process takes place. Like Vasa in *Drosophila* gametogenesis, MVH acts as a basic component of germ granules. Male germ cells with the *Mvh* null mutation do not maintain the IMC granules, and MIWI2 localization in the piP-bodies is also impaired. The loss of MIWI2 from the piP-bodies leads to a significant drop in the amount of prepachytene piRNAs. Compared with the wild-type control, the total amount of piRNAs in male germ tissues decreases by five-fold in the background of the *Mvh* mutation. A decrease in the number of piRNAs mapped to repeated DNA sequences, including transposons, is also observed [173]. MVH ATPase activity is required for MIWI2-dependent transposon silencing. Catalytic mutant *Mvh* alleles (DEAD → DQAD) cause male infertility and derepression of the *LINE1* retrotransposon, but not female sterility [174]. Activation of transposons in catalytically inactive *Mvh* mutants occurs due to a loss of prepachytene piRNAs associated with MIWI2. MILI is able to detect pre-piRNAs, bind to them, and perform slicing, but further processing with the generation of mature piRNAs is disrupted. The typical ping-pong signature in position 10 is entirely lost among MILI-linked piRNAs in catalytically inactive *Mvh* mutant males [174]. Since the nuclear localization of MIWI2 is licensed by its binding to piRNA [74], unloaded MIWI2 in fetal gonocytes of catalytically inactive *Mvh* mutants is not translocated into the nucleus [174]. This absence of MIWI2-associated piRNAs is phenocopied by that of *Mvh* null mutant males [173]. In norm, complexes of MIWI2 with piRNAs provide a control of de novo DNA methylation in genomic regions encoding transposable elements. In male mice, the methylation pattern is established in fetal gonocytes during cell cycle arrest. The DNA methylation process is disrupted by *Mili*, *Miwi2*, and *Mvh* mutations [74,173,174].

The pachytene piRNA subpopulation in the germline is the most abundant in the testes of adult males. It is known that pachytene piRNAs predominantly originate from long non-coding RNAs transcribed from intergenic regions [175]. As a rule, pachytene piRNAs are loaded into MIWI, which is expressed during the post-meiotic stages of spermatogenesis (Figure 4). According to recent studies, a significant number of mRNAs are targets of post-meiotic piRNA regulation, and the biogenesis of pachytene piRNAs occurs through the amplification ping-pong cycle [176]. There is a strict control over which mRNAs are the targets of pachytene piRNAs. When the human piRNA cluster was inserted into the mouse genome, this event caused a repression of the *Dpy192* gene, which is required for spermatogenesis in mice, resulting in male sterility [176]. The deletion of the piRNA cluster *pi18* also demonstrates that pachytene piRNAs function in the maintenance of mRNA homeostasis, which is required for the correct completion of spermatogenesis at the post-meiotic stage. Defects in the morphology of spermatozoa acrosomes and their motility are detected in such males [177]. Similar results were obtained in male mice lacking the pachytene piRNA cluster *pi6* [178].

The requirement for MVH function at the pachytene stage has also been shown using the catalytically inactive *Mvh* mutation. Germ cells of mutant males are able to proceed through meiosis but then undergo an arrest of differentiation at the round spermatid stage, followed by apoptosis. This appears to be because the mutant population of MVH protein forms irreversible complexes with precursors of pachytene piRNAs, causing a disruption of the normal dynamics of the biogenesis and the functioning of pachytene piRNAs in spermiogenesis [174], similar to what has been shown earlier for Vasa in *Bombyx mori* [66]. Thus, MVH, as a key contributor to the piRNA biogenesis and piRNA-mediated silencing, is required for the repression of mobile elements with prepachytene piRNAs associated with MIWI2 and MILI, as well as for the correct regulation of genetic elements, represented in part by protein-coding genes, with pachytene piRNAs associated with MIWI and MILI.

In addition to its involvement in the piRNA pathway, MVH is also able to interact with a specific cohort of mRNAs. MVH in the mice testes is shown to associate with several hundred mRNAs, among which a large number are responsible for spermatogenesis directly or indirectly [179]. Interestingly, MVH targets included genes encoding various translational regulators, including eIF4B, and genes involved in the energy metabolism of germ cells. Presumably, MVH selectively interacts with multiple mRNAs, providing their storage as inactive RNP complexes in CB. A release of mRNAs from these complexes and their translocation to the cytoplasm for active translation can be triggered by acetylation of MVH and decreasing its RNA-binding activity [179]. Recently published mapping of MVH binding sites in single mouse oocytes reveals overrepresented GC-rich MVH-binding motifs in target transcripts [180].

### 7.3. Post-Translational Regulation of MVH in Mice

Post-translational regulation of MVH activity is mediated by the acetylation of the lysine residue K405. The acetylated form of MVH is found at the post-meiotic stage in the CB. Histone acetyltransferase-1 (Hat1), which is found in the CB along with its cofactor p46, is responsible for MVH acetylation. This post-translational modification reduces the RNA-binding activity of MVH, while its ATP binding and ATPase activities remain unchanged [179]. How MVH acetylation modulates its functions at the molecular level remains unknown. The methylation of arginine residues within the RGG motifs in the amino-terminal domain of MVH, which is conservative in Vasa orthologues from *Drosophila* to humans, allows binding to Tudor domains of a number of protein components of germ granules and appears to regulate their structure and dynamic exchange with the cytosol [42,46,47]. Symmetric and asymmetric dimethylation of two arginine residues, R62 and R105, have been shown for MVH [47].

In recent years, studies using a mouse model have resulted in significant advances in understanding both the regulatory mechanisms of the mammalian piRNA pathway and the critical function of RNA helicase Vasa (MVH) in the piRNA silencing associated with the maintenance of spermatogenesis at different stages. The clear manifestation of sexual dimorphic functions of MVH makes the mouse the most valuable model object for studying this phenomenon. The functions of MVH as a regulator of translation of certain mRNAs are also widely investigated, with the assumption that this function is conservative in higher eukaryotes, including humans.

## 8. Human VASA (DDX4)

Germ cells of human gonads develop from a homogeneous population of PGCs by the induction mechanism. In both male and female embryonic gonads, PGCs initially proliferate, but subsequently their fates differ: female germ cells enter meiotic prophase I at the 11th week of pregnancy, and then bind to somatic cells, forming primary follicles. In comparison, the entry of male germ cells into meiosis does not occur until puberty [181,182]. Like other mammals, humans undergo a massive loss of potential oocytes before folliculogenesis by programmed cell death [92]. It was previously thought that girls are born with a limited number of resting primordial follicles, but recent research has shown that many mammalian species, including humans, have postnatal ovaries that contain mitotically active germ cells [183].

The human genome encodes a single *vasa* orthologue, *Ddx4*. Human PGCs become Vasa-positive only after colonization of the gonadal ridge of the embryo. The germline markers DAZL and Vasa (DDX4) are expressed by human fetal germ cells, but their expression patterns differ over time. In fetal ovaries, a strong increase in the expression of Vasa and DAZL mRNAs and proteins are detected in the second trimester of pregnancy, while they are not expressed in gonocytes or oogonia in the first trimester. The marked increase in Vasa expression in fetal ovaries between 9 and 14 weeks of gestation coincides with the entry of female germ cells into meiosis. In prenatal ovaries, Vasa is found in the cytoplasm of larger and more mature germ cells than DAZL and is also present in oocytes within primordial follicles [34,182,184]. In fetal oocytes at the age of 35 weeks, Vasa demonstrates a heterogeneous intracellular distribution: in addition to diffuse staining of the cytoplasm, it is also detected in the granular material located around the nucleus. This arrangement corresponds to Balbiani bodies, which were originally described as germinal complexes consisting of mitochondria, endoplasmic reticulum, and lamellar aggregates and are now considered nuage-related germinal structures [45,185,186]. In some oocytes, Vasa is not associated with the Balbiani bodies, despite having a perinuclear localization in granules. The association of Vasa with the Balbiani bodies was previously thought to be a hallmark of fetal oocytes [34,187], but this was later shown in neonatal and pubertal ovaries [188]. As germ cells progress toward meiosis entry and primordial follicle formation, the absolute number of Vasa-positive cells decreases through programmed cell death, but Vasa is easily detected in the remaining cells [187]. In neonatal and pubertal human ovaries, the Vasa protein is also detected in the cytoplasm of oocytes in early primordial follicles in a perinuclear localization corresponding to the location of the Balbiani bodies. Primordial follicles also show a subnuclear localization of Vasa, but with a broader distribution pattern compared to primordial follicles. The Vasa protein is not detected in secondary follicles or at subsequent stages of folliculogenesis [188]. The testes of a human fetus at 17 weeks of age contain one to six Vasa-positive cells per cross-section of the seminiferous tubules. A higher proportion of germ cells in fetal ovaries compared to testes corresponds to a higher level of Vasa mRNA in ovaries at the same stage [34].

The roles of Vasa and its necessity for human oogenesis and spermatogenesis remain unknown, despite the availability of data on the expression of Vasa in human gonads. Considering that Vasa-containing germ granules are present in human germ cells, it would be reasonable to suppose that Vasa functions in human gametogenesis similarly to those of other mammals. Analysis of the expression of key components of the piRNA pathway has shown that human fetal oocytes express PIWIL2 and piRNAs that are mapped to transposons, but PIWIL1 or PIWIL4 are not expressed or are below the detection threshold. While PIWI proteins are not detected in the fetal ovaries during the first trimester, PIWIL2, the mouse MILI homologue, is highly expressed later in the fetal ovaries in Vasa-positive germ cells, with PIWIL2 and Vasa both being localized to the perinuclear granules [93]. This pattern is similar to that observed in mouse gonocytes. PIWIL1 (a murine MIWI homologue), PIWIL2, and Vasa are also expressed in oocytes of adult females, but they are not found in shared granular structures [93]. Therefore, the role of Vasa in the biogenesis of human piRNAs has not been directly demonstrated by experimentation. In different organisms, *vasa/Ddx4* genetic ablation or mutations that destroy its helicase activity cause sexually dimorphic germline abnormalities, leading to either male or female sterility. Currently, there is no evidence to suggest the existence of sexual dimorphism in Vasa function in human gametogenesis. Interestingly, Vasa and its transcripts are found in the testes of adult patients with a number of disorders of spermatogenesis [189,190]. The Vasa signal in testis immunostaining preparations was found to be lower in classical seminoma and dysgerminoma than in spermatocyte seminoma. However, Vasa expression is not detected in the cases of non-seminomatous germ cell tumors, which allows for the use of Vasa as a marker for these diseases [191]. Some cases of idiopathic azoospermia or severe oligospermia are associated with hypermethylation of the Vasa promoter, which presumably reduces the expression level of its mRNA [192]. In general, the study of the functions of Vasa in human gametogenesis seems to require different approaches compared to classical model organisms such as mouse and *Drosophila*. The data obtained in mice on germ cell maintenance and spermatogenesis are not always reproducible in humans. Research on apes, such as macaques, appears to be promising, although it is very expensive [193,194].

## 9. Conclusions

A number of questions regarding the functions of Vasa and its homologues in the gametogenesis of many animals remain unanswered. Thus, the involvement of Vasa proteins in the maintenance of GSCs has been shown for *D. melanogaster* and *C. elegans* [21,97,98,159]. In all cases, however, it remains unknown whether this involvement is mediated by the piRNA pathway or different subpopulations of Vasa are required to maintain GSCs and piRNA-mediated silencing. The first assumption is supported by the need for other participants of the piRNA pathway to maintain GSCs in *Drosophila* presumably via action in the translation activation of specific mRNAs in the piRNA-dependent mode [122,123,195]. However, the involvement of Vasa in piRNA-independent regulation of translation of a number of morphogens and its direct interaction with translation initiation factors in *Drosophila* [24,98,100,101] can rather suggest the existence of functionally separated intracellular populations of Vasa. Another unresolved issue concerns the specificity of Vasa interaction with mRNA targets. It is known that conservative RecA-like domains of DEAD-box-containing helicases unwind RNA duplexes by interacting nonspecifically with the sugar–phosphate backbone of the targets [12]. Thus, the terminal domains of Vasa, which are not conservative in different animals, may be proposed to be responsible for the recognition of specific targets. The specificity of the interaction of Vasa and homologues with a number of germline transcripts has been shown for *Drosophila, C. elegans*, and mice [101,164,179,180], and some MVH-binding motifs in mRNAs have been found in mice [180]. CLIP-seq or RIP-seq libraries, with sufficient read depth, can serve as valuable material for identifying conservative and species-specific transcripts that are targets of Vasa regulation. Obtaining such data can be a difficult task, given the transient time of the processive interaction of DEAD box RNA helicases with RNAs, but the development of CLIP methods makes it possible to circumvent this problem by in vivo cross-linking of RNA-protein complexes. In some animals, specific interactions of Vasa homologues with RNA targets have not been found experimentally. It is assumed that in these cases, Vasa-like proteins in germ granules create a hub structure that receives transcripts from nucleus for unwinding, subsequent sorting, and direction into various processing pathways [196].

The greatest advances in the study of Vasa in recent years have occurred due to the discovery of the principles of organization of germ granules as liquid biocondensates phase separated from the cytosol and the basic role of Vasa proteins in their architecture and functional dynamics in a variety of metazoan species [42,48,49]. Emerging Vasa functions and mechanisms of action for ensuring gametogenesis remains to be unraveled in future studies.

## Data Availability

Not applicable.
